# X-linked lymphoproliferative disease with initial onset of neurological symptoms: a case and literature review

**DOI:** 10.3389/fimmu.2025.1677958

**Published:** 2025-10-08

**Authors:** Lijun Zhang, Deyuan Li, Jianjun Wang, Haiyang Zhang, Jun Chen, Zhongqiang Liu

**Affiliations:** ^1^ Department of Pediatrics, West China Second University Hospital, Sichuan University, Chengdu, Sichuan, China; ^2^ Key Laboratory of Birth Defects and Related Disease of Women and Children (Sichuan University), Ministry of Education, Chengdu, Sichuan, China; ^3^ Department of Pediatric Intensive Care Unit, West China Second University Hospital, Sichuan University, Chengdu, Sichuan, China

**Keywords:** X-linked lymphoproliferative syndrome, SH2D1A, central nervous system, lymphoma, immunology

## Abstract

X-linked lymphoproliferative syndrome type 1 (XLP-1) is a life-threatening X-linked recessive immunodeficiency classically characterized by susceptibility to Epstein–Barr virus (EBV), hypogammaglobulinemia, and lymphoma. While neurological involvement can occur, it is exceptionally rare as the initial and predominant manifestation. This case report details a novel presentation of XLP-1 in a 4-year-old boy who presented with acute, initial neurological symptoms (sudden fever, headache, and vomiting) in the absence of typical immune dysregulation features. Whole-exome sequencing (WES) identified a hemizygous variant in the SH2D1A gene (c.1A>G, p. Met1Val), predicted as damaging/disease-causing by MutationTaster (probability = 0.993) and PolyPhen-2 (probability = 0.992). Meanwhile, according to the American College of Medical Genetics and Genomics (ACMG) guidelines for variant interpretation, this variant met four evidence criteria (PVS1_Moderate+ +PM2_Moderate+PP3+PP5) and was classified as pathogenic. Structural analysis leveraging the AlphaFold protein structure database demonstrated that this variant disrupts the Kozak consensus sequence and splice site, critically impairing start codon recognition and translation initiation, thereby explaining the loss of functional SLAM-associated protein (SAP) protein expression. This case, along with a focused review of the literature, underscores that XLP-1 rarely presents primarily with neurological symptoms, broadening the clinical phenotype spectrum and emphasizing the need for early genetic evaluation in children with unexplained acute neurological presentations, even in the absence of overt immunodeficiency signs. This finding provides crucial clinical data for a more comprehensive understanding of XLP-1.

## 1 Introduction

X-linked lymphoproliferative syndrome (XLP) is a primary immunodeficiency disorder, classified as an X-linked recessive genetic disease, which was first discovered by Purtilo et al. in 1975 (1). Characterized by profound susceptibility to Epstein–Barr virus (EBV) infection, XLP-1 frequently precipitates life-threatening complications, including fulminant hemophagocytic lymphohistiocytosis (HLH), hypogammaglobulinemia, and malignant lymphoma (2–4). Epidemiological studies indicate an incidence of approximately one to two affected male individuals per million, with a devastating overall mortality rate of up to 75%; notably, 70% of affected individuals succumb before the age of 10 (2, 5). This high mortality is compounded by frequent diagnostic delays, as most patients present only after developing severe manifestations such as cytopenias (thrombocytopenia, anemia, pancytopenia), hepatosplenomegaly, lymphadenopathy, or lymphoma.

XLP encompasses two major subtypes: XLP-1, caused by pathogenic variants in the SH2D1A gene, and XLP-2, associated with variants in XIAP. XLP-1 is typically associated with a more severe phenotype, particularly regarding HLH and lymphoma risk. Advances in whole-exome sequencing (WES) technology have significantly enhanced the identification of underlying genetic defects in suspected inherited immunodeficiencies like XLP, enabling more precise diagnosis and facilitating early intervention strategies, including carrier screening within affected families, as demonstrated in studies like that of Woon et al. (6).

The initial presentation of XLP-1 is often non-specific and variable, commonly including fever, rash, diarrhea, and recurrent infections (3, 7). However, onset with predominant or initial central nervous system (CNS) symptoms is exceedingly rare and represents a significant diagnostic challenge. This case report details a pediatric patient with XLP-1 whose disease manifested acutely with prominent neurological symptoms, underscoring the critical importance of considering XLP-1 in the differential diagnosis of children presenting with unexplained CNS involvement, even in the absence of classic immunological features. Furthermore, we provide a focused review and analysis of previously reported XLP-1 cases with neurological onset, aiming to consolidate current understanding of this rare presentation and enhance clinical recognition.

## 2 Case report

### 2.1 Medical history and physical examination

This study has received ethical approval from the Ethics Committee of West China Second University Hospital, Sichuan University (approval no. SCMCIRB-K2020076-5). Written informed consent was obtained from the patient’s parents before conducting WES and including the patient’s clinical and imaging details in the publication.

A 4-year-old, previously healthy boy presented with a 4-day history of acute fever, headache, and vomiting. He was conscious and alert, with equal and reactive pupils bilaterally. Cranial nerve assessment (masseter strength, corneal reflexes, facial symmetry) showed normal results. Limb muscle strength and tone were normal. Crucially, no pathological reflexes or meningeal irritation signs (Kernig’s, Brudzinski’s) were present. General examination showed normal vital signs, clear lungs, regular heart sounds without murmurs, and a soft, non-tender abdomen with no organomegaly.

His history is that he was hospitalized for pneumonia 22 days before this admission. At that time, laboratory tests did not show significant lymphocyte depletion or hypogammaglobulinemia. Chest CT showed inflammation in both lungs, and the patient recovered and was discharged after antibiotic treatment. The family confirms that there is no other serious or recurrent infection history before this pneumonia. There was no recent head trauma. Parents specifically denied any family history of inherited immunodeficiencies, genetic metabolic disorders (including epilepsy), hypertension, diabetes, or obesity.

Initial assessment revealed an acutely ill child with prominent neurological symptoms (fever, headache, vomiting) in the absence of focal neurological deficits or signs of meningeal irritation. The recent history of pneumonia raised concerns but was not directly linked to the current presentation. The constellation of symptoms, particularly the acute neurological onset in a previously well young boy without a suggestive family history, prompted a comprehensive evaluation for potential infectious, inflammatory, or rarely, primary immunodeficiency disorders affecting the central nervous system.

### 2.2 Imaging and laboratory tests

Initial complete blood count was unremarkable. The level of immunoglobulin IgG was 4.94 g/L (3.41-19.6 g/L), that of IgM was 0.67 g/L (0.43-1.63 g/L), and that of IgA was 0.63 g/L (0.41-3.95 g/L). The level of CD3+ (T lymphocyte count) was 4.97 * 10^9^/L (0.90-4.50 * 10^9^/L), that of CD3+CD4+ (helper T cell count) was 2.87 * 10^9^/L (0.50-2.40 * 10^9^/L), that of CD3+CD8+ (inhibitory T cell count) was 1.65 * 10^9^/L (0.30-1.60 * 10^9^/L), that of CD19+ (B lymphocyte count) was 1.21 * 10^9^/L (0.20-2.10 * 10^9^/L), and that of CD3-CD16 + 56+ (NK lymphocyte count) was 0.49 * 10^9^/L (0.10-1.00 * 10^9^/L). Subsequent evaluation revealed key features diagnostic of HLH, including fever >38.5°C, an increase in triglyceride concentration of 3.86 mmol/L (<1.7 mmol/L), a decrease in fibrinogen of 1.26 g/L (2.0-4.0g/L), an increase in ferritin of 3871 µg/L (22-322 µg/L), and an increase in sCD25 of 4111.1 U/mL (<2,400.0 U/mL). Bone marrow smears showed a significant increase in the ratio of bone marrow to red blood cells, with obvious tissue cell phagocytosis of blood cells. It is worth noting that the patient’s EBV nucleic acid level is <200 IU/mL (<200 IU/mL), and the anti-EBV antigen IgM antibody showed a negative result. These findings are consistent with a diagnosis of non-EBV-driven HLH (**Table 1**).

**Table 1:** The main laboratory results of the patient on admission.

Items	Value	Reference range	Items	Value	Reference range
Blood routine			Pathogen		
WBC counts (10^9^/L)	6.5	4.4-11.9	Serum anti-mycoplasma IgM	Negative	Negative
Neutrophil (%)	8.9	22-65%	Serum anti-Chlamydia IgM	Negative	Negative
Hemoglobin (g/L)	104	112-149	Blood-NGS	Negative	Negative
Platelet (10^9^/L)	614	128-420	CSF-NGS	Negative	Negative
C-reactive protein (mg/L)	<0.5	0-8	Sputum examination	Negative	Negative
Procalcitonin	0.05		Sputum smear of TB	Negative	Negative
ESR (mm/H)	2	<21	Sputum culture of fungus	Negative	Negative
D-dimer (mg/L)	0.13	<0.55	Serum HIV antibody	Negative	Negative
Arterial blood gas analysis			T-spot	Negative	Negative
PH	7.4	7.35-7.45	Bone marrow biopsy	Hematophagy	Negative
PaO_2_ (mmHg)	94	75-105	Immunoglobulin		
PaCO_2_ (mmHg)	43.5	35-45	IgG (g/L)	4.94	3.41-19.6
Lymphocyte subsets			IgM (g/L)	0.67	0.43-1.63
CD3+ (10^9^/L)	4.97	0.90-4.50	IgA (g/L)	0.63	0.41-3.95
CD3+CD4+ (10^9^/L)	2.87	0.50-2.40	IgE (IU/mL)	<17.50	<60
CD3+CD8+ (10^9^/L)	1.65	0.30-1.60	C3 (g/L)	1.06	0.70-2.06
CD19+ (10^9^/L)	1.21	0.20-2.10	C4 (g/L)	0.26	0.11-0.61
CD3-CD16 + 56+ (10^9^/L)	0.49	0.10-1.00	C1q (g/L)	17.30	15.7-23.7
Blood biochemical test			Cerebrospinal fluid		
Potassium (mmol/L)	5.0	3.5-5.5	Cells (10^6^/L)	28	<15
Sodium (mmol/L)	115.3	132-146	Protein (mg/L)	5110	80-430
Urea (mmol/L)	3.7	3.2-8.2	Glucose (mmol/L)	3.72	2.5-4.5
Creatinine (mmol/L)	23	17.3-54.6	Chloride (mmol/L)	116.2	120-130
Total protein (g/L)	67.8	62-76	CSF culture	Negative	Negative
ALT (U/L)	37	<49	EBV		
AST (U/L)	38	<40	EBV-PCR (IU/mL)	<200	<200
LDH	413	120-246	EBV-IgM (U/mL)	<10	<40

WBC, white blood cell; ESR, erythrocyte sedimentation rate; ALT, alanine aminotransferase; AST, aspartate aminotransferase; LDH, lactate dehydrogenase; IgM, IgG, immunoglobulin G; immunoglobulin M; IgA, immunoglobulin A; NGS, next-generation sequencing; TB, tuberculosis; HIV, human immunodeficiency virus; PaO_2_, arterial partial pressure of oxygen; PaCO_2_, arterial partial pressure of carbon dioxide; CSF, cerebrospinal fluid; EBV, Epstein–Barr virus.

**Figure 1:**
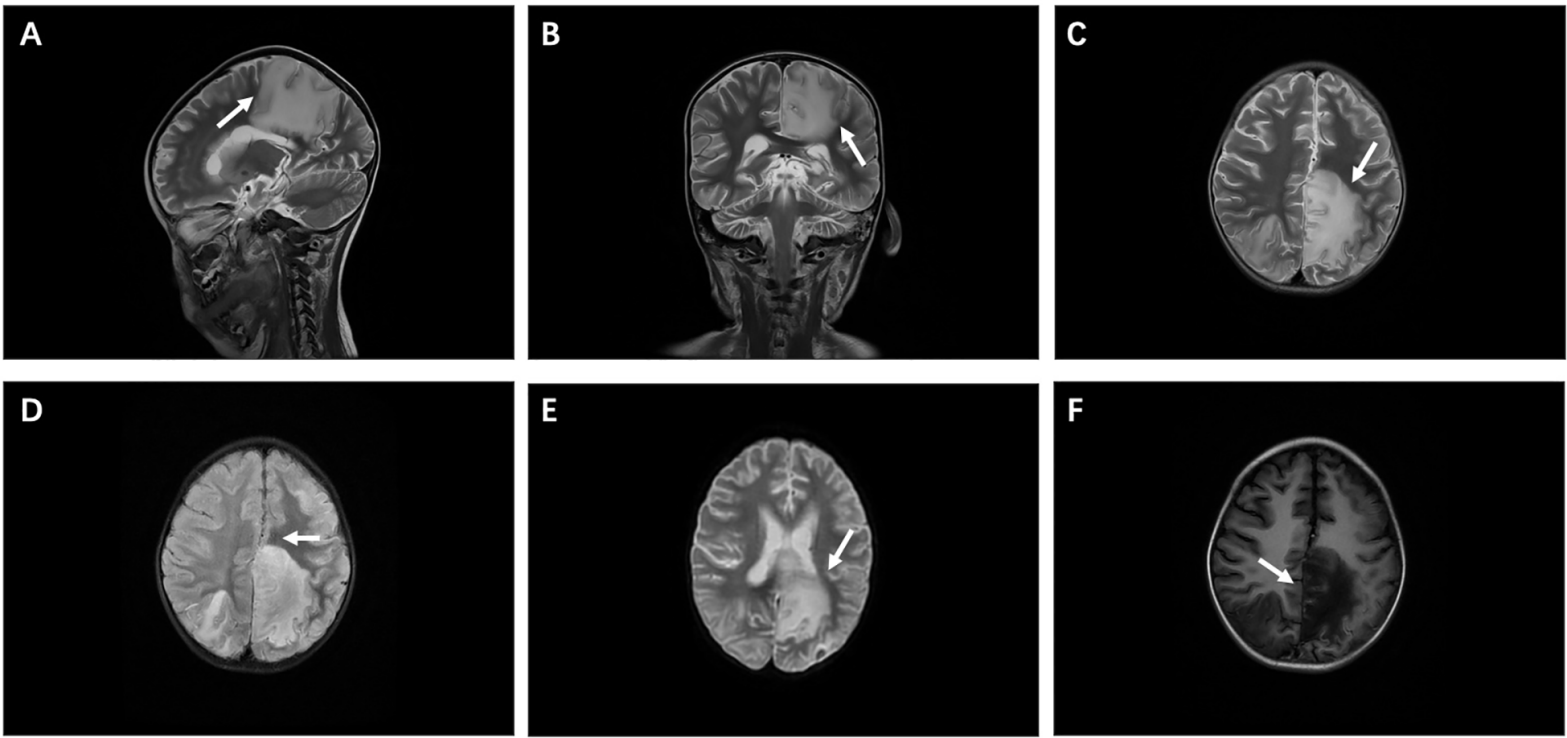
Imaging manifestations. **(A, B)** Sagittal and coronal views of cranial magnetic resonance imaging (MRI). **(C-E)** T2-weighted imaging (T2WI), fluid-attenuated inversion recovery (Flair), and diffusion-weighted imaging (DWI) show high signal intensity, mainly in the left frontal cortex and subcortical area, with the left parietal lobe reaching the deep white matter area, adjacent to the affected corpus callosum, and the midline of compression shifted to the right. **(F)** T1-weighted imaging (T1WI) shows low signal intensity.

Following admission, the patient developed central respiratory failure and acute intracranial hypertension syndrome, strongly indicating CNS involvement. Therefore, a series of examinations were conducted on the central nervous system. The patient subsequently showed increased muscle tone in the right limb and positive Babinski sign on both sides. Cerebrospinal fluid (CSF) cytology indicated a positive protein qualitative test. The number of cerebrospinal fluid cells is 28 × 10^6^/L, mainly lymphocytes, with a protein concentration of 5,110 mg/L (80–430 mg/L), a glucose concentration of 3.72 mmol/L (2.5-4.5 mmol/L), and chloride level of 116.2 mmol/L (120–130 mmol/L). No clear malignant cells were found. Basic CSF tests suggested severe impairment of the blood–brain barrier, with an increased CSF IgG synthesis index and an elevated 24-h intrathecal IgG synthesis rate. Other test results are mainly within the normal range, including cerebrospinal fluid smear, cerebrospinal fluid cryptococcal antigen, and cerebrospinal fluid culture. Cranial magnetic resonance imaging (MRI) indicated abnormal signal intensities in multiple brain regions, a nodular lesion suspicious for lymphoma in the left cerebellar hemisphere, linear leptomeningeal enhancement with associated small nodules on the left, and enhancing nodules in the right temporal lobe (**Figure 1**). Based on these findings, neuroradiology assessment raised a suspicion for intracranial lymphoma. However, definitive histopathological confirmation via biopsy was declined by the family.

Subsequently, an exhaustive diagnostic workup was undertaken to identify the etiology of the elevated intracranial pressure and multisystem involvement. Comprehensive autoimmune/rheumatologic screening (including autoantibody panels) yielded negative results. Infectious causes were systematically excluded: Tuberculosis was ruled out by negative T-SPOT and Mycobacterium tuberculosis Xpert assays; viral pathogens were excluded through negative antigen and DNA qPCR testing for a broad panel of viruses; and bacterial infection was deemed unlikely given negative blood cultures. Critically, empirical anti-infective therapy provided no clinical benefit. Given the lack of response to antimicrobials, the absence of identifiable infection or autoimmunity, the confirmed non-EBV-driven HLH, and the prominent central nervous system onset, a primary immunodeficiency disorder with multisystem inflammation, potentially due to an underlying genetic defect, became the leading diagnostic consideration.

### 2.3 Molecular examination results

**Figure 2:**
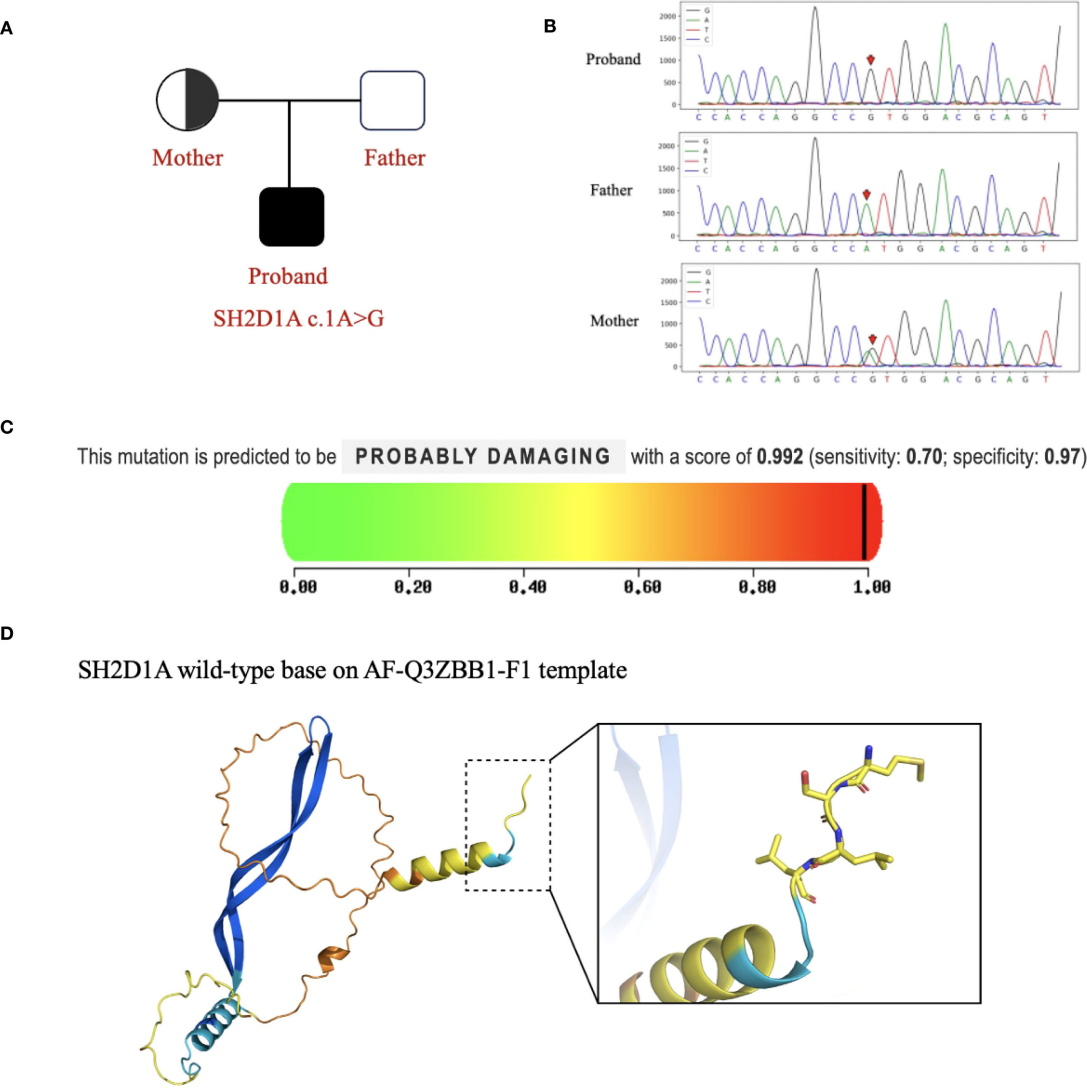
SH2D1A variants in This Family. **(A)** The proband exhibited a hemizygous variant of SH2D1A (c.1A>G). **(B)** Sequencing results of SH2D1A from specimens collected from family members. The red arrows indicate the variant site. **(C)** PolyPhen-2 predicted that the SH2D1A c.1A>G variant would be protein-damaging. **(D)** Protein structure predicted by AlphaFold and PyMOL (AF-Q3ZBB1-F1). This variant site causes changes in the Kozak consensus sequence and splice site, affecting the recognition of the start codon and translation efficiency, leading to altered protein expression.

Given the patient’s complex clinical presentation, including non-EBV-driven HLH and predominant central nervous system involvement, a primary immunodeficiency disorder of genetic origin was strongly suspected. We performed sequencing on the Illumina NovaSeq X Plus platform and used IDT xGen Exome Research Panel v1.0 for exome capture. Through paired-end 150-bp (PE150bp) mode double-ended sequencing, we produced approximately 10 G of data. The average depth of the generated raw data is >100×, and the coverage depth of the target area >20× is 95%. Finally, whole-exome sequencing (WES) identified a hemizygous variant in the SH2D1A gene (NM_002351 exon 1, c.1A>G, p. Met1Val), confirming the diagnosis of XLP-1 (**Figure 2A, B**). No other pathogenic variants potentially explaining the CNS phenotype were detected in the proband or his parents.

*In silico* analysis and structural modeling were employed to assess the variant’s pathogenicity: 1) MutationTaster predicted the variant as “disease-causing” (probability = 0.993). 2) PolyPhen-2 predicted a “probably damaging” effect on the protein (score = 0.992) (**Figure 2C**). 3) Structural modeling using the AlphaFold-predicted structure of SLAM-associated protein (SAP) (AF-Q3ZBB1-F1-template) and visualized in PyMOL (**Figure 2D**) demonstrated that the c.1A>G variant disrupts the Kozak consensus sequence and affects the splice site. This alteration is predicted to critically impair start codon recognition and translation initiation efficiency, leading to aberrant protein expression and likely a complete loss of functional SAP protein. Meanwhile, according to the American College of Medical Genetics and Genomics (ACMG) guidelines for variant interpretation (8, 9), this variant met four evidence criteria (PVS1_Moderate+ PM2_Moderate+PP3+PP5) and was classified as pathogenic. Review of previously reported SH2D1A variants underscores the rarity of our patient’s presentation: Cases with initial predominant neurological symptoms are exceedingly uncommon (7).

### 2.4 Diagnosis and treatment

Definitive diagnosis of XLP-1 was established based on clinical findings, non-EBV-driven HLH, prominent CNS involvement, and identification of a hemizygous SH2D1A variant (c.1A>G, p. Met1Val). Parental testing confirmed maternal carrier status and X-linked recessive inheritance. At the same time, the patient met the HLH-2004 diagnostic criteria (5/8 items).

Although neuroimaging strongly suggests intracranial lymphoma, the treatment team faces a major clinical dilemma: Initiating high-dose methotrexate or radiation therapy with significant neurotoxicity and bone marrow suppression toxicity is highly risky in the absence of histopathological diagnosis. In addition, the primary issue for patients is the outbreak of HLH and rapid deterioration of the nervous system, with extremely unstable vital signs, and their clinical condition cannot tolerate more invasive diagnostic procedures or intensified therapy. The most crucial thing is that the family has once again explicitly refused brain biopsies based on diagnostic or therapeutic purposes, as well as any therapies considered experimental or high-risk (**Supplementary Document 1**). Therefore, after multidisciplinary consultation, the decision is to prioritize the use of HLH-1994 protocol chemotherapy. This plan (etoposide and dexamethasone) aims to control the life-threatening HLH inflammatory storm on the one hand and also has therapeutic activity for potential lymphocytic proliferative diseases on the other hand. It is the most suitable choice under the uncertainty of diagnosis and the limitations of family willingness at that time.

Given the absence of specific pediatric guidelines and the severity of this presentation with acute neurological onset, hematopoietic stem cell transplantation (HSCT) was indicated as the only potentially curative therapy (2). Prior to planned HSCT, intensive supportive care was provided: targeted neuroprotection and intracranial pressure management, HLH-1994 protocol chemotherapy, seizure control, mechanical ventilation, and plasma exchange. The detailed treatment schedule can be found in **Supplementary Document 2**. Despite maximal intervention, reflecting the often-fatal course of XLP-1 with neurological onset (3, 7, 10), the child succumbed 2 months after symptom presentation. Autopsy was declined.

## 3 Discussion

XLP is a rare and severe primary immunodeficiency disorder, characterized by a high mortality rate due to complications such as HLH and lymphoma. The clinical management of XLP-1, a subtype of XLP caused by mutations in the SH2D1A gene, is particularly challenging due to its diverse and often atypical presentations. This report highlights the critical importance of early and accurate genetic diagnosis in patients presenting with unexplained neurological symptoms, even in the absence of typical immunodeficiency features. Our case, featuring a novel presentation of XLP-1 with acute neurological onset, underscores the innovation and clinical value of our findings.

The novelty of our case lies in that, unlike most reported cases of XLP-1 (11, 12), XLP-1 initially presents with acute, initial neurological symptoms (fever, headache, and vomiting) without concurrent EBV infection or typical immunodeficiency symptoms. The levels of IgG, IgM, and IgA in the patient are all within the normal reference range, which strongly supports atypical immune deficiency manifestations. This manifestation is extremely rare and significantly broadens the clinical phenotype spectrum of XLP-1. Our comprehensive review of previously reported cases of neurological involvement further consolidates our understanding that XLP-1 is primarily manifested in the central nervous system. By searching on PubMed with the keyword “XLP 1” from the establishment of the database until July 2025, we reviewed 248 relevant case reports and found only 10 cases, with XLP-1 presenting with initial neurological symptoms being even rarer (**Table 2**). Therefore, the lack of these “classic” markers can easily lead to clinical misdiagnosis, especially when neurological symptoms are the initial manifestation, making it difficult for doctors to immediately associate primary immunodeficiency diseases. This insight is crucial for clinical doctors as it emphasizes the necessity of early genetic assessment for children with unknown acute neurological symptoms, which may lead to early intervention and improved outcomes.

**Table 2:** Characteristics of XLP-1 patients with central nervous system involvement.

Study	Symptom	HLH (EBV)	HG	Lymphoma	Result
Hügle et al. (13)	Skin rash and high fever	Yes (+)	No	Yes	Intracranial lymphoma
Weeks et al. (14)	Night sweats, weight loss and fever	No (+)	No	Yes	Intracranial lymphoma
Liu et al. (15)	Recurrent pneumonia	Yes (-)	Yes	No	Cerebral vasculitis
Steininger et al. (12)	Recurrent respiratory infections	No (-)	No	No	Multiple cerebral aneurysms
Neves et al. (16)	Abdominal pain	No (-)	No	Yes	Cerebral vasculitis
Gray et al. (17)	Joint pain	No (-)	Yes	No	Cerebral vasculitis
Zhu et al. (18)	Headache, vomiting, and coma	No (-)	No	Yes	Cerebral vasculitis
Verhelst et al. (19)	Generalized tonic clonic seizure	No (-)	Yes	Yes	Limbic encephalitis
Dutz et al. (20)	Bronchiectasis	Yes (+)	Yes	No	Systemic vasculitis
Grosieux et al. (21)	Pityriasis lichenoid	Yes (+)	Yes	No	Extensive vasculitis

HLH, hemophagocytic lymphohistiocytosis; HG, hypogammaglobulinemia.

In addition, the patient initially exhibited severe hyponatremia (115.3 mmol/L) and extremely high cerebrospinal fluid protein (5,110 mg/L) in their cerebrospinal fluid. These findings are crucial for explaining the acute neurological manifestations of patients. Severe hyponatremia is likely a secondary phenomenon after acute central nervous system injury (such as inflammation or tumor infiltration), which may be caused by cerebral salt wasting syndrome (CSW) or antidiuretic hormone secretion disorder syndrome (SIADH) (22, 23). Hyponatremia can cause severe brain edema, significantly exacerbating symptoms such as headache, vomiting, and changes in consciousness levels. Meanwhile, extremely high levels of cerebrospinal fluid protein are a hallmark of severe disruption of the blood–brain barrier, which is fully consistent with the pathological process of severe lymphocytic inflammation or lymphoma cell infiltration in the context of XLP-1 (24). Although these secondary metabolic and barrier disorders themselves can confound clinical manifestations of the nervous system, they are not the root cause, but rather serious consequences caused by potential XLP-1-related central nervous system disorders, providing objective laboratory evidence for the severity of the condition.

The accurate diagnosis of XLP-1 is pivotal for effective clinical management, given its severe prognosis and the availability of potentially curative therapies such as HSCT. Whole-exome sequencing (WES) has emerged as a powerful tool in identifying genetic variants associated with XLP-1, offering higher accuracy and efficiency compared with traditional polymerase chain reaction (PCR) methods. In our case, WES identified a hemizygous pathogenic variant in the SH2D1A gene (c.1A>G, p. Met1Val), confirming the diagnosis. This variant, affecting the start codon and disrupting translation initiation, was predicted to be highly damaging by MutationTaster and PolyPhen-2. Our findings highlight the critical role of WES in diagnosing complex genetic disorders like XLP-1, especially when clinical presentations are atypical.

The pathophysiology of XLP-1 is intricately linked to the loss of functional SAP due to SH2D1A mutations. SAP plays a crucial role in T-cell activation and regulation of autoimmune responses. In its absence, abnormal T-cell signaling leads to severe immune dysfunction, predisposing patients to life-threatening complications such as HLH and lymphoma. Recent studies suggest that targeted therapies, such as SHP2 inhibitors, may restore T-cell function in XLP-1 patients, offering new therapeutic avenues (25). Additionally, the use of anti-CD20 antibody rituximab during acute EBV infection has shown promise in rescuing clinical symptoms, highlighting the potential benefits of preemptive B cell-targeted therapy (26). Our case, although not EBV-driven, underscores the importance of exploring such targeted therapies in the management of XLP-1.

Our case report and literature review highlight the importance of considering XLP-1 in the differential diagnosis of children presenting with unexplained neurological symptoms. The identification of the SH2D1A c.1A>G (p. Met1Val) mutation in our patient, previously reported only once (9), further emphasizes the need for genetic testing in such cases. The initial presentation with neurological symptoms, often mistaken for more common conditions like brain trauma or intracranial infections, underscores the diagnostic challenge faced by clinicians. Our findings suggest that early genetic testing and targeted therapies could significantly improve outcomes in XLP-1 patients. Future research should focus on developing comprehensive guidelines for the diagnosis and management of XLP-1, particularly in cases with atypical presentations. Multicenter, large-scale clinical studies are needed to validate the efficacy of targeted therapies and to explore novel treatment strategies. Additionally, further investigation into the pathophysiological mechanisms underlying the neurological manifestations of XLP-1 could provide new insights and therapeutic targets. A major limitation of this case is that due to the patient’s family’s refusal to undergo brain biopsy or autopsy, we are unable to obtain a clear histopathological diagnosis of intracranial space occupying lesions. Therefore, although imaging strongly suggests lymphoma, the final diagnosis is still uncertain, and other possibilities such as vasculitis or severe neuroinflammation related to HLH cannot be completely ruled out. In addition, due to limitations in experimental conditions, we did not perform routine iNKT cell count, NK cell activity, IgG subclass quantitative analysis, immunophenotyping of CSF by flow cytometry, and lymphocyte SAP staining on patients, which may be an area that we need to improve in the future.

## 4 Conclusion

In conclusion, our case report and review highlight the clinical significance and innovation in recognizing and managing XLP-1 with neurological onset. The identification of the SH2D1A c.1A>G mutation and the use of WES underscore the importance of genetic testing in diagnosing rare and complex immunodeficiency disorders. Our findings emphasize the need for early intervention and targeted therapies to improve outcomes for patients with XLP-1.

## Data Availability

The original contributions presented in the study are included in the article/**Supplementary Material**. Further inquiries can be directed to the corresponding author.
