# Supplementary figures and images for: X-linked lymphoproliferative disease with initial onset of neurological symptoms: a case and literature review
Source: Front Immunol. 2025 Oct 8;16:1677958. doi: 10.3389/fimmu.2025.1677958 (PMC12540172; doi:10.3389/fimmu.2025.1677958)

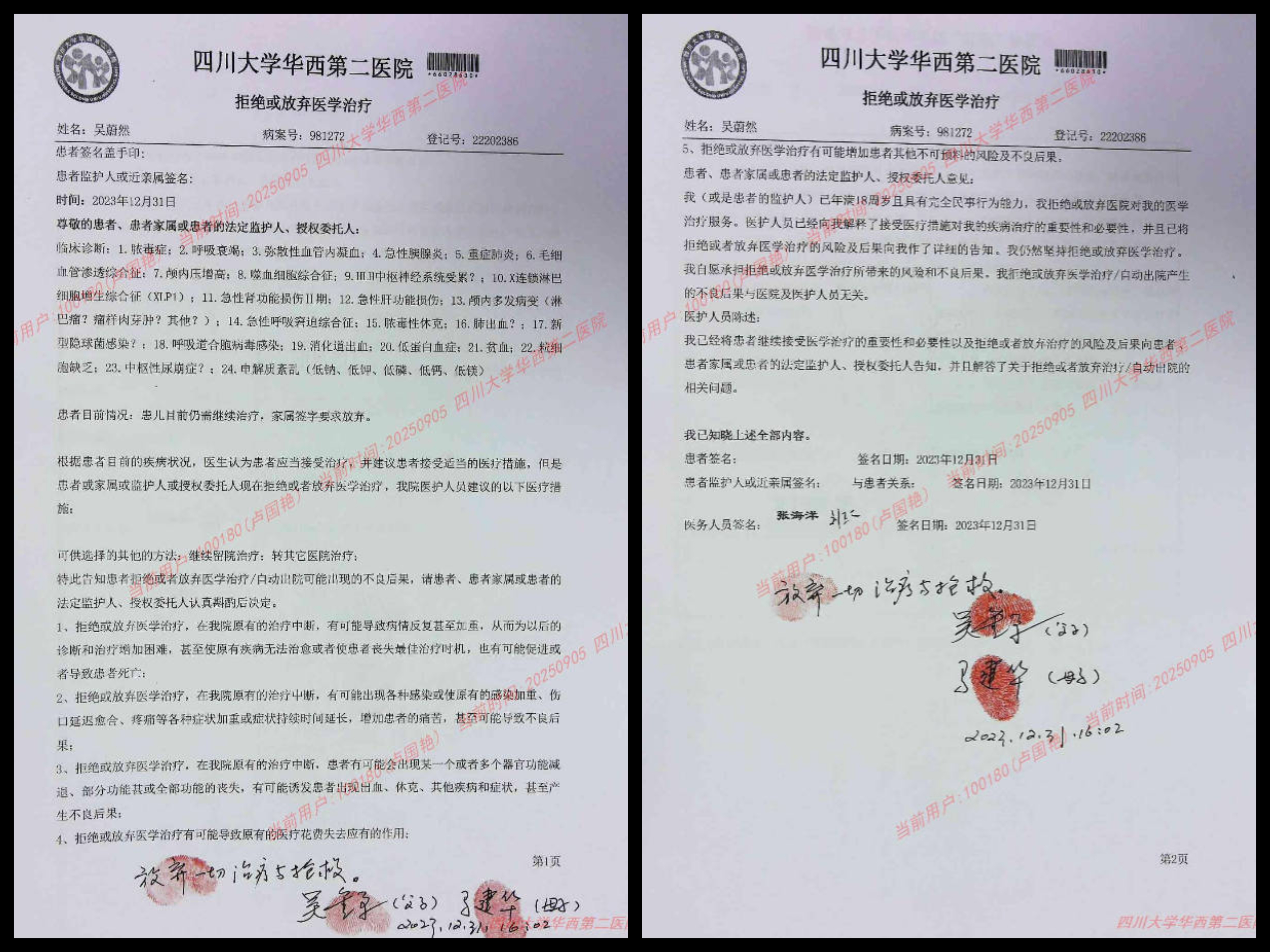

Supplement: Supplementary file 1 [file Image1.jpg]

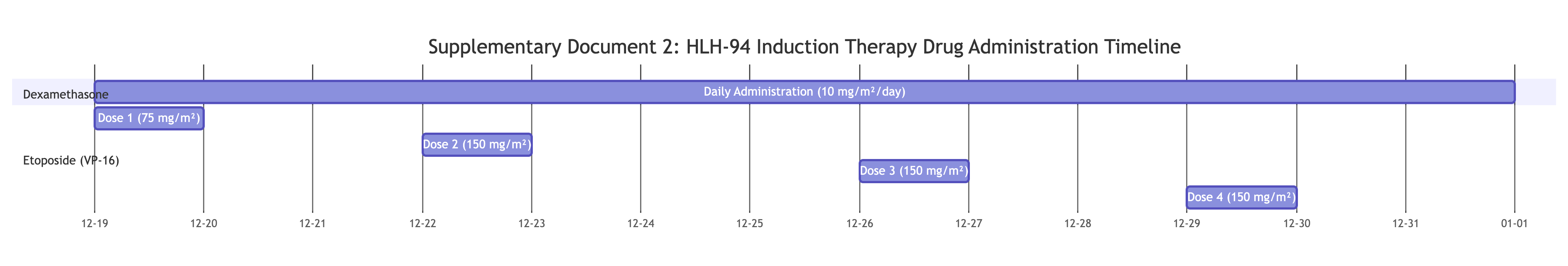

Supplement: Supplementary file 2 [file Image2.jpg]
